# Supplementary material for: Implicit Associations between Adverbs of Place and Actions in the Physical and Digital Space
Source: Brain Sci. 2021 Nov 17;11(11):1523. doi: 10.3390/brainsci11111523 (PMC8615812; doi:10.3390/brainsci11111523)
Supplement: Supplementary file 1 [file brainsci-11-01523-s001.zip › brainsci-1410124-supplementary.pdf]

brain sciences

MDPI

Article

Implicit Associations between Adverbs of Place and Actions in the Physical and Digital Space

Laila Craighero [1,*] and Maddalena Marini [2]

[1] Department of Neuroscience and Rehabilitation, University of Ferrara, 44121 Ferrara, Italy
[2] Center for Translational Neurophysiology, Istituto Italiano di Tecnologia, 44121 Ferrara, Italy; Maddalena.Marini@iit.it
* Correspondence: crh@unife.it; Tel.: +39-0532-455928

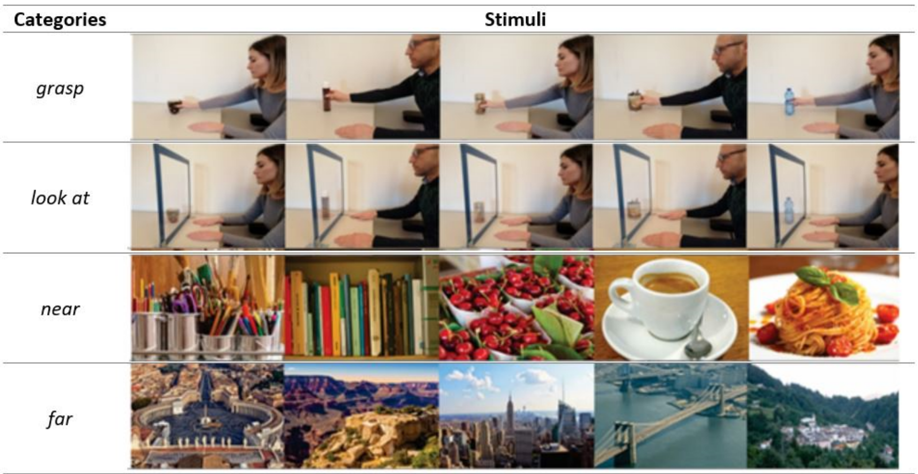

Figure S1. Double entry table presented in Experiment 1, showing images and associated categories.

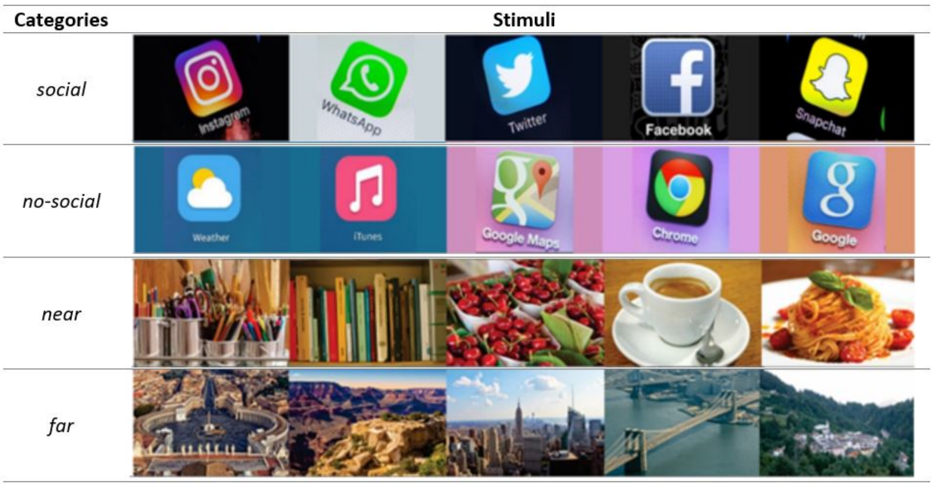

Figure S2. Double entry table presented in Experiment 2, showing images and associated categories.

Citation: Craighero, L.; Marini, M. Implicit Associations between Adverbs of Place and Actions in the Physical and Digital Space. *Brain Sci.* **2021**, *11*, 1523. https://doi.org/10.3390/brainsci11111523

Academic Editor: Peter König

Received: 21 September 2021
Accepted: 13 November 2021
Published: 17 November 2021

Publisher's Note: MDPI stays neutral with regard to jurisdictional claims in published maps and institutional affiliations.

Copyright: © 2021 by the authors. Submitted for possible open access publication under the terms and conditions of the Creative Commons Attribution (CC BY) license (https://creativecommons.org/licenses/by/4.0/).

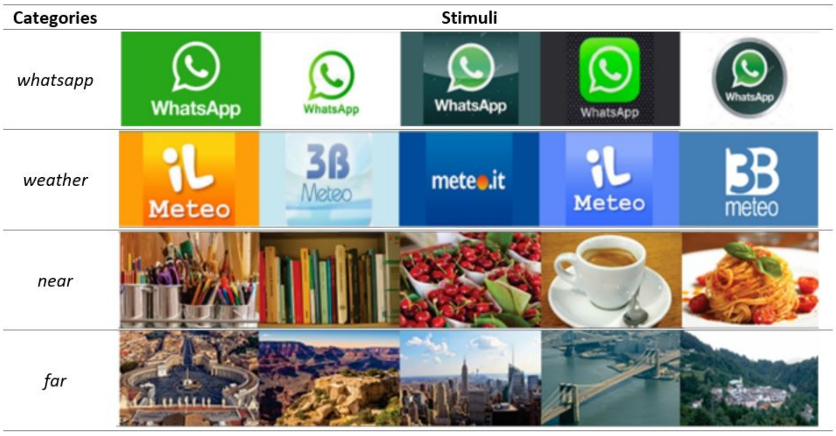

Figure S3. Double entry table presented in Experiment 3, showing images and associated categories.

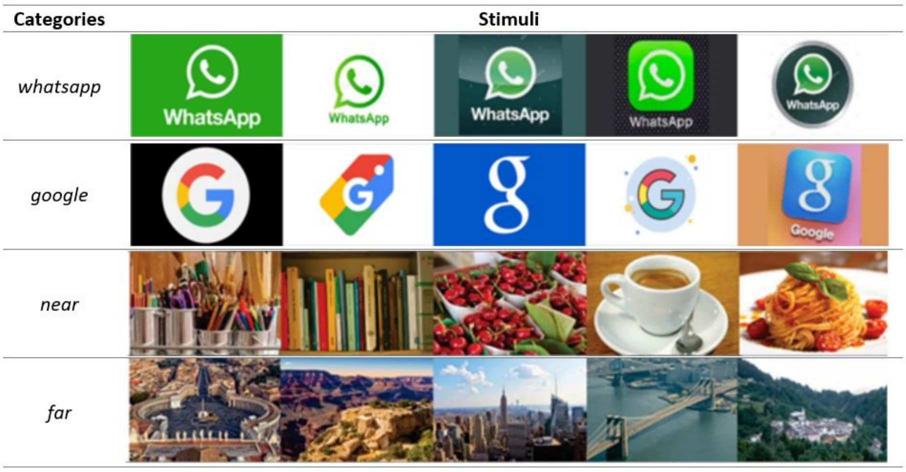

Figure S4. Double entry table presented in Experiment 4, showing images and associated categories.
